# The relationship of moral sensitivity and patient safety attitudes with nursing students’ perceptions of disclosure of patient safety incidents: A cross-sectional study

**DOI:** 10.1371/journal.pone.0227585

**Published:** 2020-01-10

**Authors:** Eunmi Lee, Yujeong Kim

**Affiliations:** 1 Department of Nursing, Hoseo University, Asan, Chungcheongnamdo, Republic of Korea; 2 College of Nursing, Kyungpook National University, Daegu, Republic of Korea; University of Adelaide, AUSTRALIA

## Abstract

Disclosure of patient safety incidents is a healthcare management strategy that primarily involves responding after incidents. We investigated the association between nursing students’ moral sensitivity, attitudes about patient safety, and perceptions of open disclosure of patient safety incidents in Korea. Data were collected from 407 nursing students at four nursing universities using self-reported moral sensitivity, attitudes about patient safety, and perceptions about open disclosure of patient safety incidents as measures. The data were analyzed using t-test, one-way analysis of variance, and a multiple regression. As moral sensitivity and attitudes about patient safety improved, nursing students’ perceptions regarding the open disclosure of patient safety incidents improved significantly. After controlling for gender, grade, and major satisfaction, the effect of changing attitudes about patient safety was greater than that of moral sensitivity for all perceptions of open disclosure. An education and intervention program is needed to improve nursing students’ attitudes about patient safety and promote the open disclosure of patient safety incidents during undergraduate training.

## Introduction

Patient safety is a major concern and global challenge in healthcare [1]. Several strategies to reduce preventable adverse events have been formulated, such as safety incident reports and organizational learning systems to learn from errors [2]. One such strategy is promoting policies that encourage disclosure of patient safety incidents. [3]. Open disclosure is a communications approach that focuses on immediate, honest disclosure to patients and families when adverse events occur in healthcare. Open disclosure includes expressing regret for what has happened and giving information to patients regarding both investigations into the adverse event and steps taken to prevent a recurrence [4]. Open disclosure maintains trust between healthcare providers and patients, reduces medical disputes, and decreases medical malpractice claims [3]. Healthcare providers favor open disclosure programs and are more likely to continue to work at institutions which encourage them [5].

Healthcare providers find communicating and expressing regret for adverse events difficult when individual fault or liability regarding the incident has not been established [6]. Nurses, who have the most contact with patients, frequently encounter various safety issues: medication errors, falls, and pressure ulcers. However, nurses experience ethical conflicts and moral stress related to openly disclosing patient safety incidents [7].

Moral sensitivity, defined as one’s ability to recognize moral conflicts, grasp the patient’s vulnerable state situationally and intuitively, and understand the consequences of healthcare decisions, is an important factor in ethical decision-making in clinical settings [8,9]. For nurses, moral sensitivity and attitudes about patient safety generally have their foundations in nursing school [10]. Along with the practical education students receive as undergraduates, they must develop positive attitudes toward and capabilities for patient safety [11]. In this way, moral sensitivity and attitudes about patient safety formed in nursing schools may continue to influence nurses’ perceptions of open disclosure in clinical settings. By examining nursing students’ moral sensitivity and attitudes about patient safety, information can be gained regarding their perceptions of open disclosure. This information can then be used to ensure timely education during undergraduate training, when professional values are being formed.

There are some studies on how doctors [12], nurses [13], and medical students [14] perceive open disclosure programs. A few studies have examined disclosure of patient safety incidents in the nursing student population. These studies have shown that nursing students believe honest communication about medical errors is valuable if it helps improve patient care [15] and that open patient safety approaches are needed so that issues can be discussed with clinical mentors [16]. There is also evidence that training can improve the ethical awareness of and the communication of patient safety incidents among nursing students [17]. During clinical practice, however, when nursing students do not receive positive feedback for openly disclosing information about patient safety incidents, they express concerns about openly disclosing incidents in the future [18,19]. Although many studies have investigated nursing students’ moral sensitivity [20], only a few have examined its relationship with attitudes about patient safety or disclosure of patient safety incidents [15,17]. Therefore, this study examines how moral sensitivity and attitudes about patient safety in nursing students affects their perceptions of disclosure of patient safety incidents. This study aims to identify how nursing students perceive disclosure of patient safety incidents; examine how general characteristics influence differences in perception of it; look for correlations between perceptions of disclosure of patient safety incidents, moral sensitivity, and attitudes about patient safety; and identify any relationship of moral sensitivity and attitudes about patient safety on perceptions of disclosure of patient safety incidents.

## Materials and methods

### Design

This cross-sectional study investigates the relationship of nursing students’ moral sensitivity and attitudes about patient safety with their perceptions of disclosure of patient safety incidents.

### Study population

This study was conducted from April 30, 2018 to June 22, 2018. Participants were recruited through campus announcements and convenience sampling at four nursing universities in Korea; the four universities were selected after considering regional coverage and the size of the nursing departments. The sample size required for this study was calculated to be 395 using G*Power 3.1, with an effect size of .02, power of .8, α of .05, and 5 predictors. Based on this calculation, we set our target sample size at 420, assuming a 5% dropout rate. Our questionnaires were distributed to 420 students; after excluding the data from 13 respondents that were inappropriate for the analysis, the remaining 407 responses were included in the final analysis.

Prior to completing the survey, the researchers explained the purpose of the study to participants in a group. The potential participants freely decided to participate in the study and provided written consent. The participants were nursing students currently enrolled in an undergraduate course who understood the purpose of the study and agreed to participate. Nursing students under 18 years old were excluded because parental consent would have been required. The survey lasted approximately 5–10 minutes per person.

### Measures

#### Demographics

The general characteristics measured were gender, age, grade, religion, economic status, major satisfaction (on a 5-point scale, where a higher score indicated greater satisfaction with the nursing major), educational experience with nursing ethics and patient safety in undergraduate courses, and familiarity with disclosure of patient safety incidents.

#### Moral sensitivity

Moral sensitivity is the ability to analyze ethical issues in the context of the ethical decision-making process, which implies contextual and intuitive understanding of the vulnerability of others’ personal situations and insight into the consequences of ethical decision-making [8]. The present study used the 27-item Korean Moral Sensitivity Questionnaire (K-MSQ), which was adapted and validated for Korean nurses by Han, Kim, Kim, and Ahn [9] from the 30-item Moral Sensitivity Questionnaire (MSQ) developed by Lutzen et al. [8]. Each item in this measure was rated on a 7-point Likert-type scale, ranging from 1 (“absolutely disagree”) to 7 (“absolutely agree”); reverse-coded items were taken into consideration. The possible score ranged from 30 to 210, with higher scores indicating higher moral sensitivity. At the time of development, the reliability coefficient in Lutzen et al. [8] was Cronbach’s α = .78, and in this study, Cronbach’s α = .823.

#### Attitudes about patient safety

Attitudes about patient safety were measured using an instrument for assessing patient safety and medical error developed by Madigosky, Headrick, Nelson, Cox, and Anderson [21]. This instrument examined patient safety knowledge, attitudes, and performance ability in healthcare students; it was modified and supplemented by Park and Park [22] to cover attitudes about patient safety. The instrument had 13 items measured on a 5-point Likert scale, with a minimum score of 13 points and maximum score of 65 points; higher scores indicate better attitudes about patient safety. Reverse-coded items were taken into consideration. In Park and Park [22], Cronbach’s ⍺ was .68, and in this study, .670.

#### Perceptions of disclosure of patient safety incidents

Nursing students’ perceptions of disclosure of patient safety incidents were measured using a questionnaire modified and supplemented by the researchers, based on Wagner et al.’s [13] study on nurses working in nursing homes, Kaldjian et al.’s [23] study on physicians, Lee et al.’s [24] study on nurses, and Ock et al’s [5] study. To test content validity and gather expert opinion on the content, the items were evaluated by four experts. The expert team was comprised of one professor from the Society for Patient Safety and three nurses specializing in patient safety with five or more years of experience. The Content Validity Index (CVI) was calculated based on the scale scores determined by the experts; the S-CVI value was .89. The questionnaire consisted of 30 items divided into 6 sub-categories, including open disclosure across harm levels (3 items), open disclosure across situations (6 items), justification of open disclosure (4 items), negative consequences of open disclosure (5 items), positive consequences of open disclosure (6 items), and facilitators of open disclosure (6 items). Each item in this measure was rated on a 4-point Likert-type scale, ranging from 1 (“absolutely disagree”) to 4 (“absolutely agree”); reverse-coded items were taken into consideration. Higher scores indicated a more favorable perception of disclosure of patient safety incidents. The reliability coefficient in this study was Cronbach’s α = .866.

### Statistical analysis

To examine the relationship of nursing students’ moral sensitivity and attitudes about patient safety on their perceptions of disclosure of patient safety incidents, it was first necessary to examine the use of control variables. Perceptions of disclosure of patient safety incidents were compared across general characteristics (gender, grade, religion, economic status, major satisfaction, educational experience on nursing ethics and patient safety, and familiarity with patient safety incident disclosure) using independent t-test and one-way analysis of variance (ANOVA). Age and major satisfaction were analyzed using a multiple regression analysis to compare perceptions of disclosure of patient safety incidents.

Correlations between perceptions of disclosure of patient safety incidents, moral sensitivity, and attitudes about patient safety were examined. To study the relationship of nursing students’ moral sensitivity and attitudes about patient safety on their perceptions of open disclosure, a multiple regression analysis was conducted with perceptions of open disclosure (six sub-categories) as the dependent variables, moral sensitivity and attitudes about patient safety as independent variables and general characteristics that differ on perceptions of open disclosure by each category as control variables.

This paper was written following STROBE guidelines for the reporting of cross-sectional studies [25].

### Ethics approval and consent to participate

This study was approved by the Hoseo University Institutional Review Board (No. 1041231-180424-HR-076-01). Ethical issues regarding plagiarism, informed consent, misconduct, data fabrication and/or falsification, double publication and/or submission, and redundancy have been addressed fully by the author. Appropriate measures were taken to protect participants against coercion or unjust influence during the recruitment or consent process. Only those who voluntarily agreed to participate could fill out the written informed consent and questionnaire. An explanation of the research purposes and the questionnaire was provided. It was made clear to the students that they had the freedom to not participate in this research, that there were no advantages or disadvantages resulting from their participation, and that they could stop at any time. The participation agreement included a statement confirming participants’ anonymity and confidentiality.

## Results

### General characteristics of participants

Of the 407 respondents, 354 (87.0%) were female (mean age = 21.57 years); 210 (51.6%) were freshmen or sophomores; 235 (57.7%) had no religion; 320 (78.6%) described themselves as middle class economically; 237 (58.3%) had high satisfaction with their major (mean satisfaction score = 3.63 on a 5-point Likert scale); 345 (85.0%) were educated about nursing ethics in their undergraduate courses; 214 (52.7%) were educated about patient safety, and 101 (24.9%) had heard of open disclosure (Table 1).

**Table 1:** General characteristics of participants (n = 407).

	Categories		n (%), Mean±SD	
Gender		Male		53 (13.0)
		Female		354 (87.0)
Age				21.57±2.03
Grade		Years 1–2		210 (51.6)
		Years 3–4		197 (48.4)
Religion		No		235 (57.7)
		Yes		172 (42.3)
Economic status		Low		47 (11.5)
		Middle		320 (78.6)
		High		40 (9.8)
Major satisfaction		Very low		2 (0.5)
		Low		19 (4.7)
		Moderate		149 (36.6)
		High		196 (48.2)
		Very high		41 (10.1)
		Mean		3.63±0.75
Received nursing ethics education		No		61 (15.0)
		Yes		345 (85.0)
Received patient safety education		No		192 (47.3)
		Yes		214 (52.7)
Familiarity with open disclosure		No		305 (75.1)
		Yes		101 (24.9)

### Perceptions of disclosure of patient safety incidents

Mean responses were calculated for each item measuring perceptions of disclosure of patient safety incidents (Table 2). In the category of open disclosure across harm levels, the statement “In the event of a medical error causing serious harm, healthcare providers should notify the patients and families” had the highest mean score at 3.70 (range 1–4). In the category of open disclosure across situations, the statement “Patient safety incidents should be disclosed even when the healthcare providers determine that the patient or families would not fully understand the explanation” had the highest mean score at 3.43. In the category of justification of open disclosure, all four items had similar mean scores, from 3.56 to 3.66. In the category of negative consequences of open disclosure, all five items had mean scores below 3; “Open disclosure of patient safety incidents will increase medical litigation” had the lowest mean score at 2.16. In the category of positive consequences of open disclosure, except for “Open disclosure of patient safety incidents will reduce the healthcare providers’ sense of guilt,” all items had mean scores above 3. In the category of facilitators of open disclosure, “It is necessary to provide guidelines for open disclosure of patient safety incidents” had the highest mean score at 3.64.

**Table 2:** Perceptions of disclosure of patient safety incidents.

Categories	Item	Mean±SD
Open disclosure across harm levels	In the event of a medical error causing serious harm, healthcare providers should notify the patients and families.	3.70±0.48
	In the event of a medical error causing minor harm, healthcare providers should notify the patients and families.	3.42±0.60
	In the event of a medical error causing no harm, healthcare providers should notify the patients and families.	3.00±0.79
Open disclosure across situations	Patient safety incidents should be disclosed even when the healthcare providers determine that the patient or families would not fully understand the explanation.	3.43±0.59
	Patient safety incidents should be disclosed even when the healthcare providers determine that the patients or families would not want to know about the incident.	3.20±0.70
	Patient safety incidents should be disclosed even when the healthcare providers determine that the patients or families would not find out about the incident.	3.34±0.64
	Patient safety incidents should be disclosed even when the healthcare providers determine that it would not be beneficial for the patient or families to find out about the incident.	3.20±0.69
	Open disclosure of patient safety incidents should be determined based on the severity of the medical error.*	2.28±0.93
	Patient safety incidents should be disclosed based on whether informing the patient or families about the medical error would benefit them.*	2.25±0.89
Justification of open disclosure	I think apologizing for a patient safety incident is important in my values.	3.65±0.54
	It is necessary to disclose patient safety incidents because the patient would want to know about patient safety incidents.	3.66±0.51
	Open disclosure of patient safety incidents is needed even if it causes loss and disadvantage for the hospital and healthcare providers.	3.56±0.54
	Healthcare providers have the responsibility to inform the patients and families about their or their team’s errors.	3.59±0.51
Negative consequences of open disclosure	Patients and families will react negatively to disclosure of patient safety incidents.*	2.44±0.78
	Open disclosure of patient safety incidents will increase medical litigation.*	2.16±0.72
	Open disclosure of patient safety incidents will damage the reputation of the healthcare providers.*	2.71±0.78
	Healthcare providers will be subject to disciplinary actions by healthcare institutions if they disclose patient safety incidents.*	2.32±0.71
	Healthcare providers will be subject to criticism by their colleagues if they disclose patient safety incidents.*	2.70±0.77
Positive consequences of open disclosure	Patients and families will have more trust in healthcare providers who disclose patient safety incidents.	3.18±0.74
	They are more likely to recommend to others around them healthcare providers who disclose patient safety incidents.	3.16±0.73
	They are more likely to return for treatment to healthcare providers who disclose patient safety incidents.	3.17±0.73
	Healthcare providers who disclose patient safety incidents are more likely to provide better services.	3.42±0.63
	Open disclosure of patient safety incidents will lead healthcare providers themselves to be more interested in patient safety issues.	3.58±0.52
	Open disclosure of patient safety incidents will reduce the healthcare providers’ sense of guilt.	2.89±0.80
Facilitators of open disclosure	Open disclosure of patient safety incidents requires higher ethical awareness by the healthcare providers.	3.63±0.51
	Education and training programs are needed for open disclosure of patient safety incidents.	3.62±0.52
	Healthcare institutions need personnel to support open disclosure of patient safety incidents.	3.52±0.58
	Healthcare institutions need a positive culture of patient safety that supports open disclosure of patient safety incidents.	3.62±0.52
	It is necessary to provide guidelines for open disclosure of patient safety incidents.	3.64±0.51
	It is necessary to establish apology laws to protect healthcare providers.	3.46±0.62

* Reverse-coded items

### Differences in perceptions of disclosure of patient safety incidents based on general characteristics

In terms of examining the differences in perceptions of disclosure of patient safety incidents, the relationship between the control and dependent variables only showed a statistically significant difference when considering satisfaction levels with the student’s major. Higher major satisfaction indicated a more favorable perception of disclosure of patient safety incidents (F = 2.700, p = 0.030). In terms of examining the differences in the six sub-categories of perceptions of disclosure of patient safety incidents, there were statistically significant differences in open disclosure across harm levels and negative consequences of open disclosure across grade (F = 2.249, p = 0.025 / F = 2.261, p = 0.025), in positive consequences of open disclosure across grade (F = 2.060, p = 0.040) and major satisfaction (F = 2.892, p = 0.022), and in facilitators of open disclosure across gender (F = -2.260, p = 0.024) and major satisfaction (F = 3.886, p = 0.004). Open disclosure across situations and justification of open disclosure did not show a statistically significant difference for any of the general characteristics (Table 3).

**Table 3:** Differences in perceptions of disclosure of patient safety incidents based on general characteristics.

		Total perceptions of disclosure of patient safety incidents		Open disclosure across harm levels		Open disclosure across situations		Justification of open disclosure		Negative consequences of open disclosure		Positive consequences of open disclosure		Facilitators of open disclosure	
		Mean±SD, slope	t, F	Mean±SD, slope	t, F	Mean±SD, slope	t, F	Mean±SD, slope	t, F	Mean±SD, slope	t, F	Mean±SD, slope	t, F	Mean±SD, slope	t, F
Gender	Male	95.25±9.75	-0.233 (0.816)	10.11±1.59	-0.075 (0.943)	17.91±3.31	0.513 (0.610)	14.23±1.97	-0.955 (0.270)	12.72±2.53	1.097 (0.247)	19.53±2.56	0.390 (0.697)	20.75±2.51	-2.260 (0.024)
	Female	95.55±8.88		10.13±1.51		17.66±2.70		14.50±1.61		12.28±2.75		19.38±2.67		21.61±2.59	
Age		0.188	0.855 (0.393)	-0.024	-0.637 (0.525)	0.100	1.474 (0.141)	0.031	0.768 (0.443)	-0.040	-0.602 (0.548)	0.072	1.110 (0.268)	0.048	0.756 (0.450)
Grade	Years 1–2	96.13±8.95	1.439 (0.151)	10.29±1.50	2.249 (0.025)	17.91±2.71	1.622 (0.106)	14.36±1.68	-1.313 (0.190)	12.63±2.52	2.261 (0.025)	19.66±2.57	2.060 (0.040)	21.29±2.51	-1.698 (0.090)
	Years 3–4	94.85±9.00		9.95±1.52		17.46±2.86		14.57±1.64		12.02±2.90		19.12±2.72		21.73±2.66	
Religion	No	95.30±8.88	-0.554 (0.580)	10.13±1.59	-0.002 (0.999)	17.57±2.72	-1.038 (0.304)	14.37±1.67	-1.241 (0.215)	12.37±2.49	0.267 (0.790)	19.49±2.60	0.871 (0.384)	21.37±2.68	-1.193 (0.234)
	Yes	95.80±9.14		10.13±1.42		17.86±2.87		14.58±1.65		12.29±3.03		19.26±2.73		21.68±2.47	
Economic status	Low	95.83±7.96	2.524 (0.081)	10.38±1.33	2.593 (0.076)	17.51±2.65	1.571 (0.209)	14.81±1.53	1.182 (0.308)	11.91±2.69	1.024 (0.360)	19.62±2.40	2.285 (0.103)	21.60±2.56	1.289 (0.277)
	Middle	95.10±8.96		10.04±1.51		17.63±2.76		14.41±1.68		12.34±2.75		19.27±2.64		21.41±2.62	
	High	98.45±9.90		10.53±1.66		18.43±3.09		14.48±1.71		12.75±2.59		20.18±2.93		22.10±2.36	
Major satisfaction	Very low^a^	99.50±10.61	2.700 (0.030) b < e	11.00±1.41	1.100 (0.356)	20.00±1.41	0.637 (0.636)	15.50±0.71	2.360 (0.053)	11.00±5.66	1.246 (0.291)	19.50±4.95	2.892 (0.022) b < e	22.50±2.12	3.886 (0.004) c < e
	Low^b^	94.42±10.48		9.74±1.56		17.42±3.50		14.84±1.26		11.21±3.05		19.16±3.82		22.05±2.55	
	Moderate^c^	94.80±9.19		10.20±1.46		17.56±2.67		14.31±1.69		12.42±2.53		19.32±2.52		20.99±2.81	
	High^d^	95.25±8.63		10.04±1.54		17.72±2.80		14.40±1.73		12.30±2.58		19.20±2.52		21.59±2.49	
	Very high^e^	99.68±8.36		10.41±1.56		18.05±2.87		15.10±1.22		12.78±3.66		20.71±2.84		22.63±1.74	
	Mean	1.298	2.189 (0.029)	0.046	0.456 (0.649)	0.133	0.721 (0.471)	0.124	1.126 (0.261)	0.250	1.384 (0.167)	0.322	1.833 (0.068)	0.423	2.478 (0.014)
Received nursing ethics education	No	94.80±8.85	-0.665 (0.506)	10.14±1.52	0.127 (0.899)	17.53±2.92	-1.136 (0.257)	14.38±1.73	-0.981 (0.329)	12.28±2.60	-0.410 (0.681)	19.24±2.69	-1.092 (0.276)	21.42±2.59	-0.539 (0.590)
	Yes	95.63±9.03		10.12±1.51		17.84±2.67		14.54±1.61		12.39±2.84		19.53±2.62		21.56±2.60	
Received patient safety education	No	94.98±9.03	-1.115 (0.265)	9.82±1.47	-1.742 (0.078)	17.59±2.23	-0.309 (0.713)	14.21±1.75	-1.260 (0.231)	12.05±2.93	-0.903 (0.397)	19.59±2.73	0.633 (0.537)	21.54±2.91	0.150 (0.881)
	Yes	95.98±8.96		10.19±1.52		17.71±2.88		14.50±1.65		12.39±2.69		19.36±2.65		21.49±2.54	
Familiarity with open disclosure	No	95.34±8.75	1.230 (0.298)	10.17±1.47	0.979 (0.357)	17.74±2.84	0.592 (0.554)	14.49±1.62	0.639 (0.523)	12.26±2.64	-0.686 (0.493)	19.25±2.57	-1.747 (0.098)	21.43±2.57	-0.930 (0.362)
	Yes	95.88±9.63		10.00±1.65		17.55±2.62		14.37±1.80		12.48±2.89		19.78±2.84		21.70±2.66	

### Correlations between perceptions of disclosure of patient safety incidents, moral sensitivity, and attitudes about patient safety

Perceptions of disclosure of patient safety incidents (30–120) had a mean score of 95.51. Mean scores for each sub-category were as follows: 10.13 for open disclosure across harm levels (3–12), 17.69 for open disclosure across situations (6–24), 14.46 for justification of open disclosure (4–16), 12.33 for negative consequences of open disclosure (5–20), 19.40 for positive consequences of open disclosure (6–24), and 21.50 for facilitators of open disclosure (6–24). The mean moral sensitivity score (30–210) was 154.92, and the mean attitudes about patient safety score (13–75) was 50.84. There were statistically significant positive correlations between all variables, whereas negative consequences of open disclosure did not show statistically significant correlations with justification of open disclosure, facilitators of open disclosure, or moral sensitivity (Table 4).

**Table 4:** Correlations between perceptions of disclosure of patient safety incidents, moral sensitivity, and attitude about patient safety.

	Cronbach’s α	Mean	SD	Min	Max	Pearson’s r							
						Overall perceptions of disclosure of patient safety incidents	Open disclosure across harm levels	Open disclosure across situations	Justification of open disclosure	Negative consequences of open disclosure	Positive consequences of open disclosure	Facilitators of open disclosure	Moral sensitivity
Total perceptions of disclosure of patient safety incidents	0.866	95.51	8.98	69	120								
Open disclosure across harm levels	0.701	10.13	1.51	5	12								
Open disclosure across situations	0.682	17.69	2.79	10	24		0.438 (<0.001)						
Justification of open disclosure	0.809	14.46	1.66	8	16		0.506 (<0.001)	0.437 (<0.001)					
Negative consequences of open disclosure	0.773	12.33	2.73	5	20		0.203 (<0.001)	0.204 (<0.001)	0.077 (0.122)				
Positive consequences of open disclosure	0.712	19.40	2.65	12	24		0.326 (<0.001)	0.276 (<0.001)	0.375 (<0.001)	0.304 (<0.001)			
Facilitators of open disclosure	0.885	21.50	2.59	11	24		0.270 (<0.001)	0.313 (<0.001)	0.537 (<0.001)	-0.016 (0.746)	0.396 (<0.001)		
Moral sensitivity	0.823	154.92	14.75	116	195	0.320 (<0.001)	0.150 (0.002)	0.117 (0.019)	0.345 (<0.001)	-0.024 (0.625)	0.316 (<0.001)	0.375 (<0.001)	
Attitude about patient safety	0.670	50.84	5.15	39	64	0.496 (<0.001)	0.308 (<0.001)	0.293 (<0.001)	0.492 (<0.001)	0.157 (0.002)	0.273 (<0.001)	0.463 (<0.001)	0.494 (<0.001)

### Effect of moral sensitivity and attitudes about patient safety on perceptions of disclosure of patient safety incidents

To analyze the effect of nursing students’ moral sensitivity and attitudes about patient safety on their perceptions of disclosure of patient safety incidents, two regression models were used (Table 5). Model 1 analyzed the effect of moral sensitivity and attitudes about patient safety on perceptions of disclosure of patient safety incidents. Statistically significant results were found for all perceptions of disclosure of patient safety incidents (overall: F = 68.509, p < 0.001, R^2^ = 0.253; open disclosure across harm levels: F = 21.149, p < 0.001, R^2^ = 0.095; open disclosure across situations: F = 19.273, p < 0.001, R^2^ = 0.087; justification of open disclosure: F = 69.389, p < 0.001, R^2^ = 0.256; negative consequences of open disclosure: F = 8.026, p < 0.001, R^2^ = 0.038; positive consequences of open disclosure: F = 27.060, p < 0.001, R^2^ = 0.118; facilitators of open disclosure: F = 64.809, p < 0.001, R^2^ = 0.243).

**Table 5:** Effects of moral sensitivity and attitude about patient safety on perceptions of disclosure of patient safety incidents.

		Total perceptions of open disclosure		Open disclosure across harm levels		Open disclosure across situations		Justification of open disclosure		Negative consequences of open disclosure		Positive consequences of open disclosure		Facilitators of open disclosure	
		β, R^2^	t, F (p)	β, R^2^	t, F (p)	β, R^2^	t, F (p)	β, R^2^	t, F (p)	β, R^2^	t, F (p)	β, R^2^	t, F (p)	β, R^2^	t, F (p)
Model 1	Moral sensitivity	0.099	2.001 (0.046)	-0.003	-0.051 (0.960)	-0.037	-0.684 (0.494)	0.136	2.752 (0.006)	-0.134	-2.396 (0.017)	0.240	4.474 (<0.001)	0.193	3.872 (<0.001)
	Attitude about patient safety	0.447	9.043 (<0.001)	0.309	5.681 (<0.001)	0.312	5.704 (<0.001)	0.425	8.604 (<0.001)	0.223	3.975 (<0.001)	0.154	2.871 (0.004)	0.368	7.400 (<0.001)
	Model fit	0.253	68.509 (<0.001)	0.095	21.149 (<0.001)	0.087	19.273 (<0.001)	0.256	69.389 (<0.001)	0.038	8.026 (<0.001)	0.118	27.060 (<0.001)	0.243	64.809 (<0.001)
Model 2	Moral sensitivity	0.108	2.144 (0.033)	0.018	0.326 (0.745)	-0.030	-0.532 (0.595)	0.140	2.772 (0.006)	-0.139	-2.433 (0.015)	0.256	4.682 (<0.001)	0.191	3.769 (<0.001)
	Attitude about patient safety	0.442	8.907 (<0.001)	0.307	5.626 (<0.001)	0.313	5.707 (<0.001)	0.423	8.514 (<0.001)	0.223	3.980 (<0.001)	0.149	2.767 (0.006)	0.357	7.177 (<0.001)
	Model fit	0.263	28.570 (<0.001)	0.110	9.913 (<0.001)	0.097	8.578 (<0.001)	0.260	28.172 (<0.001)	0.058	4.912 (<0.001)	0.137	12.718 (<0.001)	0.259	28.040 (<0.001)

Model 1 control variable: none

Model 2 control variables: gender, grade, major satisfaction

Model 2 analyzed the effect of moral sensitivity and attitudes about patient safety on perceptions of disclosure of patient safety incidents after including the control variables. There were no differences in the results for perception of disclosure of patient safety incidents, with or without the use of control variables. Statistically significant results were found for all perceptions of open disclosure (overall: F = 28.570, p < 0.001, R^2^ = 0.263; open disclosure across harm levels: F = 9.913, p < 0.001, R^2^ = 0.110; open disclosure across situations: F = 8.578, p < 0.001, R^2^ = 0.097; justification of open disclosure: F = 28.172, p < 0.001, R^2^ = 0.260; negative consequences of open disclosure: F = 4.912, p < 0.001, R^2^ = 0.058; positive consequences of open disclosure: F = 12.718, p < 0.001, R^2^ = 0.137; facilitators of open disclosure: F = 28.040, p < 0.001, R^2^ = 0.259). The effect of attitudes about patient safety was greater than that of moral sensitivity for all perceptions of open disclosure except for positive consequences of open disclosure. The relationships of moral sensitivity with open disclosure across harm levels and across situations were not statistically significant.

## Discussion

In this study, despite the fact that only around 25% of participants were familiar with open disclosure, the perception of disclosure of patient safety incidents was generally positive. These results are similar to those found in previous studies [13,26,27]. The results of this study differ from those of the previous studies in terms of how likely participants were to determine the importance of open disclosure based on the severity of the incident in question and whether the information would be helpful to the patient and their family. In this study, nursing students’ perception of disclosure of patient safety incidents was found to be similar to that of Korean doctors, nurses, and the general public in previous studies [24,28]. Responses regarding negative consequences of open disclosure showed low scores, implying that nursing students may be more reluctant to disclose patient safety incidents based on the severity of the incident, that is, if incidents are less severe or less harmful. Their reluctance appears to originate with worries about the negative effects of open disclosure; they do not appear to believe that disclosure which does not benefit the patient or family is necessary. A study of registered nurses and registered practical nurses in Canadian nursing homes [13] likewise showed significantly higher intention to disclose to physicians and family members when the adverse event caused significant harm. Research in China and Japan has shown that nurses experience ethical and moral dilemmas due to conflict between patients’ rights and the need to protect themselves, their colleagues, and the institution [29,30]. In other studies, open disclosure of medical errors has been shown to cause healthcare providers concern regarding potential reputation damage, conflict, and litigation [13,28]. Creating a positive culture that encourages reporting of patient safety events should help alleviate the psychological burden experienced by healthcare providers [26]. Healthcare institutions must establish a positive culture where openly disclosing patient safety incidents is viewed first and foremost as creating opportunities to actively improve hospital safety. The public and the media must also create an atmosphere that demonstrates trust and support for healthcare providers and institutions in disclosing patient safety incidents. This will allow prospective healthcare providers, such as nursing students, to develop positive perceptions of disclosure of patient safety incidents.

Attitudes about patient safety, moral sensitivity, and perceptions of disclosure of patient safety incidents were positively correlated. Specifically, when patient safety attitude was positive, perceptions of disclosure of patient safety incidents were more favorable. The regression analysis also showed that patient safety attitude had a significant relationship with perceptions of disclosure of patient safety incidents. Research on nursing college students has reported that patient safety attitude affects patient safety management performance [22]. Research on nurses has also shown that the safety attitude of healthcare providers has the greatest influence on safety activities [31]. Patient safety training programs have been found to improve healthcare provider attitudes towards patient safety, which indicates that teaching this information to nursing students should also improve their attitudes towards patient safety. However, the study showed no significant difference in perceptions of open disclosure between groups receiving and not receiving patient safety education. Currently, patient safety education in Korea is not organized as part of the regular nursing education curriculum, but only briefly mentioned under the nursing management subject [32]. More curriculum development will be necessary for detailed, professional education and training in undergraduate nursing courses. In another study, healthcare providers thought that they should disclose patient safety incidents that caused harm, but also believed that they lacked the necessary experience, knowledge of methods, and ability for disclosure [33]. In the US, the Quality and Safety Education for Nurses (QSEN) initiative was launched to incorporate patient safety content in the nursing curriculum [34], including using role-playing and other techniques to teach providers how to support open disclosure [21]. In Europe, standardized guidelines for patient safety education are devised through the European Network for Patient Safety (EUNetPaS) project [35]. In Finland, the Finnish Patient Safety Strategy for 2009 to 2013 emphasizes the need for patient safety education, including undergraduate nursing education [36]. In Korea, guidelines and educational programs for disclosing patient safety events, adapted to the circumstances of the Korean medical field, are yet to be developed [37]. Korean undergraduate nursing programs must strengthen patient safety education and foster the ability to detect and solve patient safety issues, as well as the ability to empathize and communicate effectively with patients and families in the event of a patient safety incident.

Here, the relationship of moral sensitivity with perception of disclosure of patient safety incidents was significant regardless of controlling for other variables. However, several studies have reported that nurse respondents felt they could not take actions even though they believed it was ethically appropriate to do so [25,38]. Nurses experienced significant moral stress when their uncertainty about their roles as nurses and their legal responsibilities conflicted with the decision of disclosure of patient safety incidents [39]. Sufficiently effective disclosure of patient safety incidents may not be achieved by only appealing to individual ethical responsibilities and emphasizing moral justification [24]; therefore, proactive management of organizational ethics in healthcare organizations is imperative and important [40]. Creating a culture of patient safety, establishing open policies and guidelines, continuing training, and support for healthcare professionals can improve the trust relationship between patients and healthcare providers [41,42] and enable all of them to learn more from unexpected patient safety incidents [43]. Thus, is necessary to provide an institutional system and policies that can support an environment in which patient safety accidents can be disclosed and discussed effectively in Korea.

### Limitations

This study has several limitations. First, although the questionnaire was conducted anonymously and nursing students enrolled in the researchers’ own schools were excluded, social desirability bias might have occurred in self-reporting of perceptions or attitudes, leading results on moral sensitivity, attitudes about patient safety, and perceptions of disclosure of patient safety incidents to have been overestimated. Second, because this is a cross-sectional study, it is difficult to precisely define the causality between variables. A longitudinal study is needed to examine whether attitudes about patient safety, moral sensitivity, and other factors potentially influencing perceptions of disclosure of patient safety incidents change before and after clinical practice or after working as a nurse after graduation.

## Conclusion

This study examined perceptions of disclosure of patient safety incidents, moral sensitivity, and attitudes about patient safety among nursing students, matters which were unexplored in previous studies. The results highlight the importance of attitudes about patient safety and moral sensitivity for perceptions of disclosure of patient safety incidents. Patient safety training should start during undergraduate nursing education, and the curriculum should include training which will foster moral sensitivity and help construct positive patient safety attitudes. This study may serve as a justification for urgent implementation of training programs, development of standardized guidelines. By taking these measures, open disclosure can be more widely and successfully implemented, allowing healthcare providers, patients, and families to protect and support each other.

## Supporting information

S1 File (XLSX)
